# Different limb lengths in gastric bypass surgery: study protocol for a Swiss multicenter randomized controlled trial (SLIM)

**DOI:** 10.1186/s13063-021-05313-6

**Published:** 2021-05-19

**Authors:** Marko Kraljević, Romano Schneider, Bettina Wölnerhanssen, Marco Bueter, Tarik Delko, Ralph Peterli

**Affiliations:** 1 Clarunis, Department of Visceral Surgery, University Center for Gastrointestinal and Liver Diseases, St. Clara Hospital and University Hospital Basel, 4002 Basel, Switzerland; 2 St. Clara Research Ltd, Kleinriehenstrasse 30, 4058 Basel, Switzerland; 3 Department of Visceral Surgery, University Hospital Zurich, Rämistrasse 100, 8091 Zürich, Switzerland

**Keywords:** Randomized controlled trial, Roux-en-Y gastric bypass, Different limb lengths, Weight loss

## Abstract

**Background:**

Obesity and type 2 diabetes mellitus are reaching epidemic proportions. In morbidly obese patients, bariatric operations lead to sustained weight loss and relief of comorbidities in the majority of patients. Laparoscopic Roux-Y-gastric bypass (RYGB) is one of the most frequently performed operations, but it is still unknown why some patients respond better than others. Therefore, a number of variations of this operation have been introduced. Recent evidence suggests that a longer bypassed biliopancreatic limb (BPL) has the potential to be more effective compared to the standard RYGB with a shorter BPL length. This article describes the design and protocol of a randomized controlled trial comparing the outcome of a RYGB operation with a long versus short BPL.

**Methods/design:**

The trial is designed as a multicenter, randomized, patient- and observer-blinded trial. The relevant ethics committee has approved the trial protocol. To demonstrate that long BPL RYGB is superior compared to short BPL RYGB in terms of weight loss and resolution of T2DM, the study is conducted as a superiority trial. Postoperative percent total weight loss and nutritional deficiency rate are the primary endpoints, whereas morbidity, mortality, remission of obesity-related comorbidities and quality of life are secondary endpoints. Eight hundred patients, between 18 and 65 years and with a body mass index (BMI) from 35 to 60 kg/m^2^ who meet the regulatory rules for bariatric surgery in Switzerland, will be randomized. The endpoints and baseline measurements will be assessed pre-, intra-, and postoperatively.

**Discussion:**

With its high number of patients and a 5-year follow-up, this study will answer questions about effectiveness and safety of long BPL RYGB and provide level I evidence for improvement of the standard RYGB. These findings might therefore potentially influence global bariatric surgery guidelines.

**Trial registration:**

ClinicalTrials.gov NCT04219787. Registered on 7 January 2020.

**Supplementary Information:**

The online version contains supplementary material available at 10.1186/s13063-021-05313-6.

## Background

The rising prevalence of morbid obesity is causing a major health burden in terms of morbidity and mortality [1]. Complications of obesity, especially type 2 diabetes mellitus (T2DM), are placing a growing demand on healthcare resources. The prevalence of T2DM increases parallel to obesity and currently; there are 55.2 million people with T2DM in Europe, accounting for 8.5% of the adult population [2]. About half of the obesity-associated healthcare costs in Switzerland are attributed to diabetes [3]. Morbid obesity is a chronic disease for which multimodal therapeutic management strategies are necessary, comparable to cancer treatment, to reach partial or full remission with a persistent risk of relapse and thus, long-lasting follow-up is mandatory. Medical therapeutic strategies (diet, behavioral changes, or drugs) to achieve and maintain clinically significant weight loss remain limited [4].

Bariatric surgery is currently the most effective treatment for morbid obesity [4–7]. Although laparoscopic sleeve gastrectomy has become the most commonly performed bariatric procedure worldwide [8], laparoscopic Roux-en-Y gastric bypass (RYGB) is the most frequently performed bariatric procedure in Switzerland. From a scientific point of view, it is no longer a question if this procedure has a significant effect on metabolic control, but rather how the outcomes after RYGB can be further improved.

Since its introduction in 1967 by Mason [9], there have been many multiple technical variations of the RYGB to increase weight loss. Most studies used an alimentary limb (AL) length of 100 to 150 cm and a biliopancreatic limb (BPL) length of 50 to 120 cm, while the common limb (CL) length remained not measured [10, 11].

Scopinaro et al. [12] developed the biliopancreatic diversion (BPD) technique in 1979 and concluded that the BPD seems to be the most powerful treatment for hyperlipidemia and T2DM. Available evidence suggests that the extended BPL length in patients after BPD may be one of the key factors explaining the superiority of this procedure, which is further supported by some observational studies reporting greater weight loss in patients after RYGB with a longer BPL [13–15].

To this day, only one randomized controlled trial (RCT) [16] compared long BPL RYGB (150 cm BPL, AL 75 cm) with a short BPL RYGB (BPL 75 cm, AL 150 cm) in 128 patients and found a significant increase in percent excess weight loss (%EWL) for patients with long BPL RYGB in the first 4 years after surgery. However, the underlying mechanism of a greater weight loss after long BPL RYGB remains unclear. Furthermore, lengthening of the BPL cannot be done limitless as it carries an increasing risk of severe malnutrition. Neither of the two procedures seems to be technically more challenging, since the complication rates did not show any significant difference [16]. However, a limitation of this study was the cohort of 146, which was too small to show any advantage in terms of comorbidities resolution or quality of life changes.

Therefore, our aim is to investigate if a longer BPL in RYGB leads to greater weight loss and superior remission of comorbidities in morbidly obese patients without reducing its safety. No matter which outcome will be obtained, our results will provide level I evidence for an improvement of the standard laparoscopic RYGB and thus may potentially influence global bariatric surgery guidelines.

## Methods/design

The overall objective of this study is to evaluate whether long BPL RYGB is superior compared to short BPL RYGB in treating morbid obesity and the associated comorbidities.

### Primary objective

The primary objective of this study is to show that long BPL leads to a greater percent total weight loss (%TWL, superiority), while not leading to a larger nutritional deficiency rate (non-inferiority) at 1-, 3-, and 5-year follow-up.

### Secondary objective

The secondary objectives are to assess the percent excess body mass index loss (%EBMIL), remission of comorbidities, complication rate/safety, and quality of life 1, 3, and 5 years after long and short BPL RYGB.

### Study design and site

**Fig. 1:**
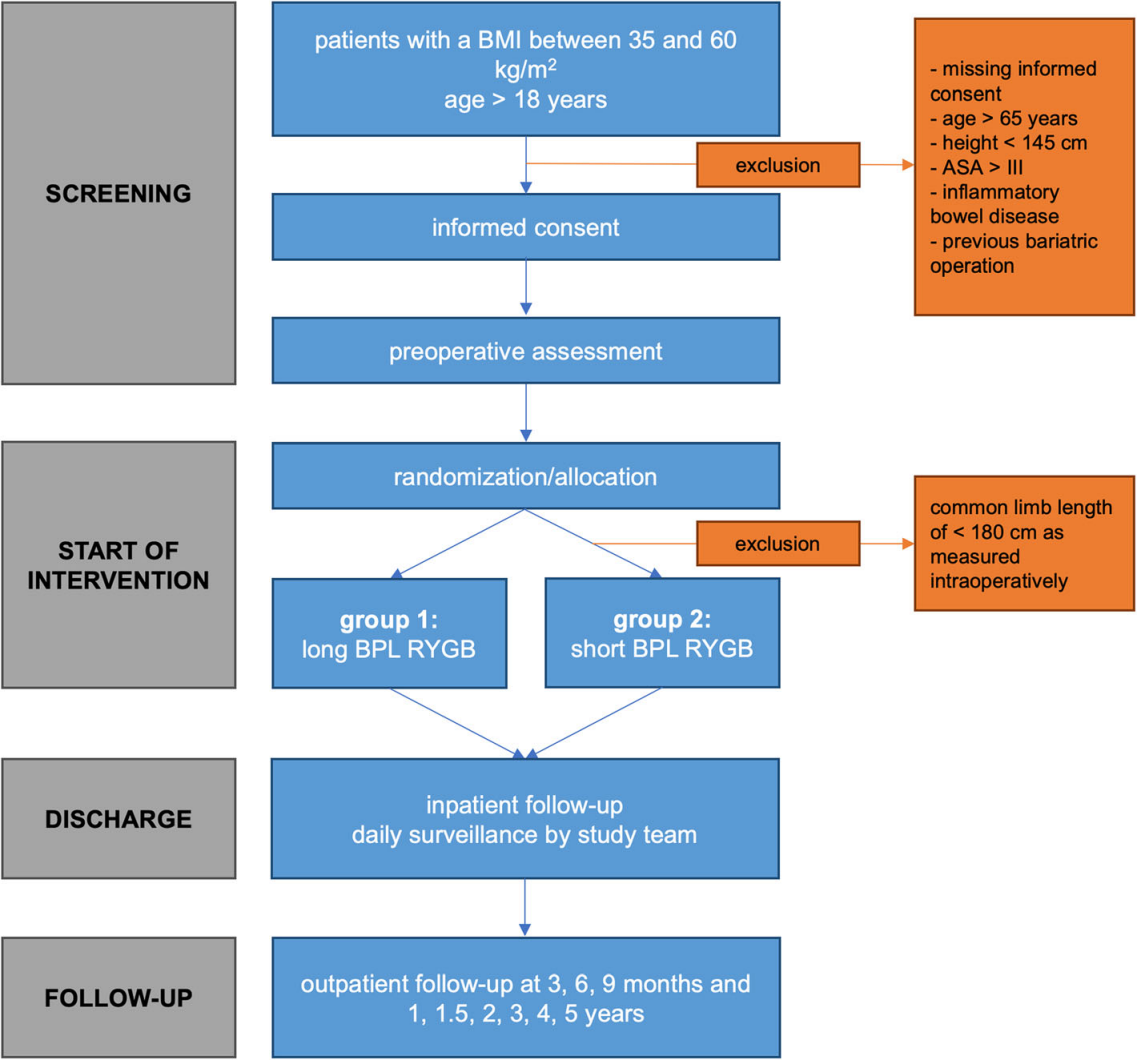
Schedule of enrollment, interventions, and assessments according to the Standard Protocol Items: Recommendations for Interventional Trials (SPIRIT) guideline

This is a multicenter, randomized, controlled, patient- and observer-blinded, superiority trial in morbidly obese patients receiving either long BPL or short BPL RYGB. All participating centers (4 university hospitals and 11 cantonal, regional, and private hospitals) have knowledge and experience in bariatric surgery and were certified as Bariatric Reference Centers by the Swiss Society for the Study of Morbid Obesity and Metabolic Disorders (SMOB). The trial has been registered on ClinicalTrials.gov under the identifier NCT04219787. This protocol has been written in accordance with the Standard Protocol Items: Recommendations for Interventional Trials (SPIRIT) guidelines (Additional file 1). The planned visit and examination schedule is presented in Fig. 1.

### Inclusion criteria

Participants fulfilling the following criteria are eligible for the study:

- Informed consent (IC) as documented by signature (Appendix Informed Consent Form)
- Patients with BMI of 35 kg/m^2^ or higher who comply with the regulatory rules for bariatric surgery in Switzerland (SMOB guidelines [17])

### Exclusion criteria

The presence of any one of the following exclusion criteria will lead to exclusion of the participant:

- Age < 18 years or > 65 years
- BMI > 60 kg/m^2^
- Height < 145 cm
- CL length of < 180 cm as measured intraoperatively
- ASA physical status classification > III
- Previous bariatric operation
- Inflammatory bowel disease
- Ongoing malignant disease
- Known or suspected non-compliance, drug or alcohol abuse
- Psychiatric disorder

### Randomization

**Fig. 2:**
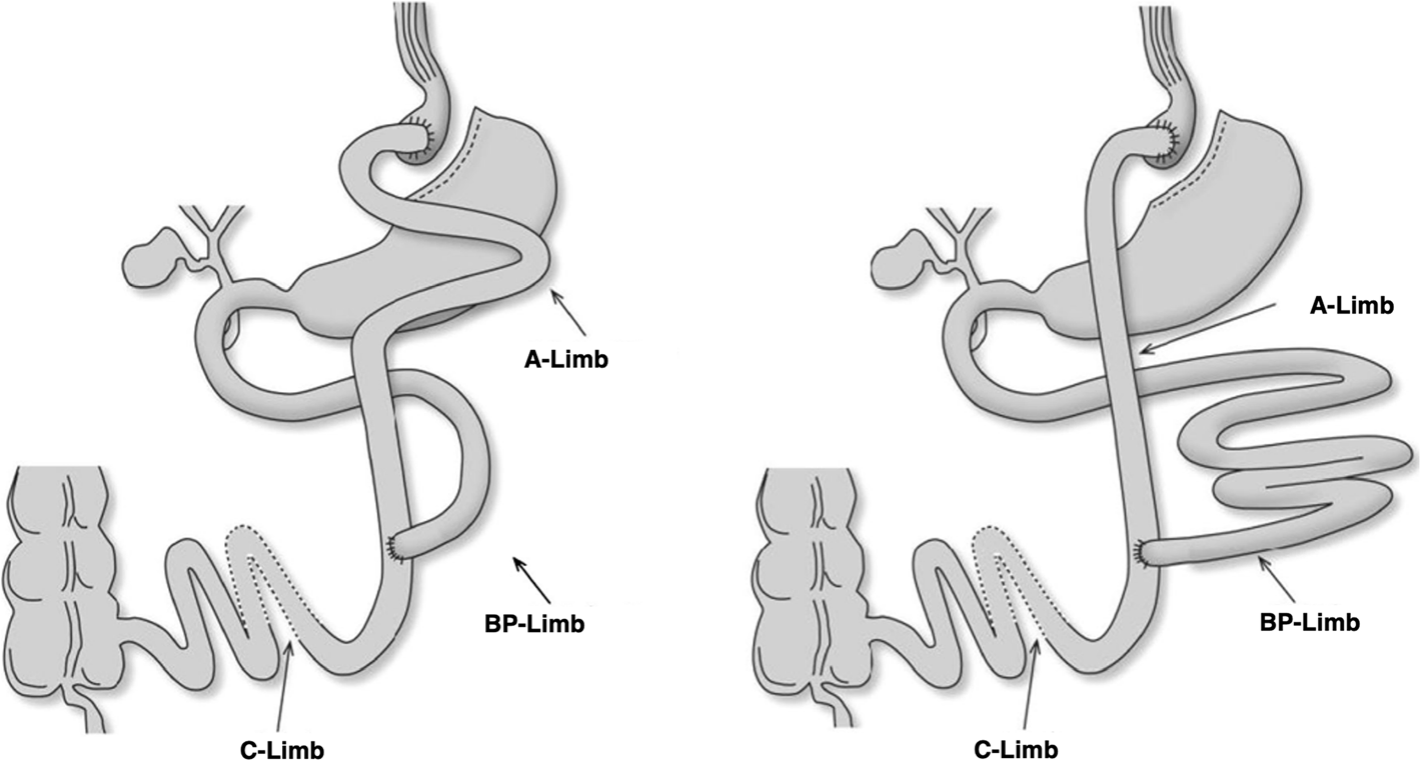
Enrolled patients receive a Roux-en-Y gastric bypass either with long biliopancreatic limb (BP-limb) and short alimentary limb (A-limb) or short biliopancreatic limb and long alimentary limb (A-limb). The common limb (C-limb) should remain the same for both groups

Eligible patients, who give written confirmed consent, will be registered in the electronic data capture and management system secuTrial®, which is programmed by the Clinical Trial Unit (CTU) of the University of Basel. The database is web-based and allows online randomization stratified by the involved centers. An allocation ratio of 1:1 with a block size of 4 will ensure a balance in sample size across both groups over time. The randomization allocation will only be known to the CTU staff. Patients will be enrolled and randomly assigned by the surgeon to receive a long BPL RYGB (arm A) versus a short BPL RYGB (arm B). Figure 2 provides a schematic view of both types of RYGBs.

### Interventions

All patients will receive standard preoperative assessment including endocrine, pulmonary function and cardiovascular assessment, psychiatric assessment, gastroscopy with Helicobacter pylori testing, and abdominal ultrasound to check for liver size and gallstones. Patients in arm A will receive a RYGB with a 180-cm-long BPL and an AL of 80 cm; the group B will receive a RYGB with an 80-cm BPL and a 180-cm AL. The individual technique in terms of trocar position, materials used (i.e., staplers, trocar, suture) will be left to the choice of each individual surgeon. Total small bowel length will be measured during surgery; patients with a total small bowel length of less than 440 cm will be excluded from the study. Patient- and observer-blinded pre- and postoperative follow-up will be performed. There are no strategies for patients to improve adherence to intervention protocol since this is a surgical trial. All participating centers and surgeons are experienced bariatric surgeons with a minimal total caseload of more than 200 bariatric procedures and more than 50 surgeries per year. In order to make surgeons adhere to intervention protocol, all critical surgical steps (e.g., pouch size, total bowel length measurement, technique of Roux-limb and gastroenterostomy construction, closure of mesenteric defects, and operation time) will be documented in our database. The non-adherent cases will be excluded from per-protocol analysis. There will be no criteria for modifying the operative procedures.

### Study visits

Physicians blinded to the intervention will perform study documentation and patient assessment. Since the trial is designed as an observer and patient blinded RCT, information about the surgical procedure will not be disclosed during the follow-up examinations to any assessors. There will be 11 study visits in total. The study design flow diagram is presented in Table 1. Potential study participants will be identified by the preoperative outpatient clinic consultations. The first visit will be preoperative after informed consent is obtained. Then patients will receive their study intervention according to the preoperative randomization. Postoperatively, study visits will be performed at discharge, 3, 6, 9, 12, and 18 months and 2, 3, 4, and 5 years. Each study visit includes data collection of weight, blood tests (e.g., hemoglobin, creatinine, urea, uric acid, calcium, C-reactive protein, albumin, liver function tests, fasting glucose, HbA1c, vitamins A, B1, B6, B12, and D, parathormone, ferritin, folate, zinc, cholesterol, lipoproteins, and triglycerides), and complications. In addition, questionnaires will be also assessed to study the quality of life (Bariatric Analysis and Reporting Outcome System [BAROS] [18] and the Gastrointestinal Quality of Life Index [GIQLI] [19]), the Arts questionnaire [20] to determine postoperative determine dumping syndrome and questionnaire for quantification of frequency and severity of potential diarrhea.

**Table 1:** Schedule of study assessments

Study periods	Screening	Intervention	Follow-up								
Visit	1	2	3	4	5	6	7	8	9	10	11
Time (day, month, year)	− 3 months	0–5 days	3 months	6 months	9 months	1 year	1.5 years	2 years	3 years	4 years	5 years
Patient information and informed consent	x										
Demographics	x										
Medical history	x										
Inclusion/exclusion criteria	x	x									
Physical examination	x	x	x	x	x	x	x	x	x	x	x
Vital signs	x	x	x	x	x	x	x	x	x	x	x
Laboratory tests	x	x	x	x	x	x	x	x	x	x	x
Randomization		x									
Operation		x									
Complications		x	x	x	x	x	x	x	x	x	x
Primary variables	x	x	x	x	x	x	x	x	x	x	x
Secondary variables	x	x	x	x	x	x	x	x	x	x	x
Questionnaires	x			x		x		x	x	x	x
Adverse events		x	x	x	x	x	x	x	x	x	x

### Study endpoints

#### Primary endpoint

The primary endpoints are %TWL and the number of nutritional deficiencies 1, 3, and 5 years postoperatively. For the %TWL the presurgical weight (at hospital entry time of the patient) and postsurgical weight at the corresponding visit will be measured.

#### Secondary endpoints

- %EBMIL
- Remission of comorbidities
- Complication rate
- Quality of life

All comorbidities will be assessed at 3, 6, 9, 12, and 18 months and 2, 3, 4, and 5 years by a physician based on symptoms, laboratory findings, and use of medication. The postoperative course of comorbidities will be defined as follows: remission: no symptoms/without any medication (dyslipidemia true remission = no medication and normal lipid values); remission of T2DM will be defined according to the ADA criteria: complete remission: HbA1c < 6.0%, fasting glucose < 100 mg/dl and at least one year no active pharmacologic therapy; partial remission HbA1c < 6.5% [21]; improvement: less symptoms and/or less medical treatment/medication; unchanged: same symptoms and equivalent therapy; worsened: more symptoms or increase of therapy; de novo comorbidity: comorbidity not present at baseline, but newly developed within 5 years postoperatively. All surgical and non-surgical complications will be assessed according to the Clavien-Dindo classification (CDC) [22] and the Comprehensive Complication Index (CCI) [23]. The Arts score will help to identify potential dumping syndrome. BAROS and GIQLI scores will be used to measure postoperative quality of life.

### Blinding

With exception of the team in the operating theater, all involved medical and non-medical practitioners are blinded as well as the patient. The procedure will be named as *laparoscopic Roux-en-Y gastric bypass* in all medical records without mentioning the limb lengths. Unblinding is permitted at the end of the study and in case of surgical or medical complications, emergency consultation, or other ethical considerations. The operating surgeon will not be involved in any decisions that influence the primary endpoint or in judgement of the primary endpoint. Furthermore, the efficacy of blinding measures will be regularly assessed according to by Probst et al. [24].

### Study management and administration

Data management and monitoring is supported by the CTU of the University of Basel. Source data of every study participant are entered into the study data management system secuTrial® (interActive Systems GmbH, Berlin, Germany).

### Quality control measures

Continuous central and on-site monitoring of the study is performed by the CTU for quality control and assurance purposes to evaluate the progress of the study and to verify the accuracy and completeness of eCRFs. All assessors will be trained in data entry to ensure data quality. Data entered into the eCRF will be validated for completeness and discrepancies automatically. Furthermore, the data will be reviewed by the responsible investigator as well as by an independent monitor. Furthermore, the CTU will ensure that all protocol requirements are met, and all applicable local authority regulations and investigator’s obligations are being fulfilled and to resolve any inconsistency in the study records. Monitoring will consist of one initiation visit, one monitoring visit per year, and a close-out visit per center as a minimum.

### Statistical analyses

#### Sample size

Our study was designed to evaluate whether long BPL in RYGB has advantages compared to short BPL in terms of % weight loss (superiority) without compromising the safety in terms of nutritional deficiency rate (non-inferiority) at 5 years follow-up. For our primary outcome efficacy and safety, assuming a %TWL of 27 ± 10% at 5 years follow-up for short BPL with a minimal improvement of 5% TWL for long BPL to be relevant and an expected cumulative nutritional deficiency rate of 50% at 5 years follow-up with a non-inferiority margin defined at 10%, 400 patients per group will be needed to reach a power of 80% with an alpha level at 5%. For further details on calculations, see the “Analysis of endpoints” section below.

#### Analysis of endpoints

The study results will be reported in adherence to the extension of the CONSORT statement from 2010 on reporting of randomized trials [25]. Summary statistics will be used to describe and compare patient characteristics of all suitable, but non-included patients and all included patients (overall and stratified for the two treatment arms). Study endpoints will be analyzed for the intention-to-treat (ITT) population and the per-protocol (PP) population. Sensitivity analysis will be conducted for the ITT population. Thereby, the ITT population includes all randomized patients in the groups to which they were randomly assigned, regardless of their adherence with the entry criteria, regardless of the treatment they actually received, and regardless of subsequent withdrawal or deviation from the protocol. In the PP population, all protocol violators, including anyone who switched groups or missed measurements, are excluded. For each group, a number of participants (denominator) included in each analysis and whether the analysis was by original assigned groups will be given. Additional sensitivity analysis will be used if, despite all efforts taken to ensure complete data collection, the number of missing data is non-negligible or could potentially bias the results and conclusions.

#### Analysis of primary endpoint

The primary objective consists of two separate analyses, one for safety and one for efficacy. The difference in efficacy is tested by performing a Mann-Whitney *U* test, thereby releasing the assumption of normality for the proportion of weight loss. The proportion of safety events during the first 5 years in each study arm is reported, including the two-sided 95% confidence interval.

#### Analysis of secondary endpoints

To investigate superiority of the experimental treatment in the long-term, analysis used for the primary endpoint will be repeated for %EBMIL at 1, 3, and 5 years postoperatively. The number of peri- and post-surgical complications will be analyzed by the chi-squared or Fisher’s exact test dependent on the CCI score. The other secondary endpoints will be investigated using appropriate explorative methods and graphical visualization.

### Ethical considerations

Participation in this trial is strictly voluntary and patients are allowed to exit the trial at any point without explanation. All eligible patients are provided an information brochure describing the study with sufficient information for them to make an informed decision about their participation in this study.

The study protocol, patients’ information sheets and informed consents were approved by the local ethic committee (Ethikkommission Nordwest- und Zentralschweiz, EKNZ 2019-02392). In addition, insurance coverage for general liability has been obtained.

Patients who decline to participate in this study are treated according to clinical standards. These patients will not be included and no study-specific follow-up will be performed.

### Participants’ confidentiality

The participants’ confidentiality is maintained at all times. For confidentiality reasons, electronic case report forms (eCRF) do not contain any personal data of study participants. Members of the ethics committees are obliged to respect confidentiality and to refrain from divulging the participants’ identity or any other personal information they might be aware of. Source data in the hospital’s electronic patient information systems are secured by personal passwords and handled with respect to medical secrecy.

### Archiving and data retention

The investigator will maintain all study-related records, such as eCRFs, medical records, laboratory reports, informed consent documents, safety reports, information regarding participants who discontinued, and other pertinent data. All records will be retained by the investigator as long as required by the applicable laws and regulatory requirements (10 years). Thereafter, all data will be destroyed. The study is conducted in compliance with this protocol and according to Good Clinical Practice standards as well as legal regulations. Direct access to source documents will be permitted for purposes of monitoring, audits, and inspections.

### Dissemination policy

The results of the study will be published in a peer-reviewed medical journal and presented at national and international scientific conferences. Authorships will be based on the recommendations of the International Committee of Medical Journal Editors.

## Discussion

The positive effects of bariatric surgery on weight loss, obesity-related comorbidities, and mortality have been widely demonstrated in long-term cohort trials and short-term RCTs [26, 27]. Over time, these procedures have improved in respect to safety and can be offered at a low mortality and morbidity [28]. The sleeve gastrectomy and the RYGB are the most common surgical procedures [8]. However, the laparoscopic RYGB is still the most commonly performed bariatric procedure in Switzerland [17]. It is no longer a question, if the RYGB has a significant effect on weight loss and on obesity-associated comorbidities, but rather how to improve the procedure and its outcomes.

The RYGB is a complex anatomical concept with various parts for potential improvement including the limb lengths. For many years, the length of the AL was thought to be the most influential factor regarding postoperative weight loss. Therefore, previous studies focused on the effects of various AL lengths, until Choban et al. showed that lengthening the AL has no effect on weight loss [29]. In contrast, the effect of the BPL length on weight loss has been studied to a much lesser extent. While the non-randomized study by Leifsson et al. reported excellent weight loss in patients with long BPL [14], only one RCT addressed this question after RYGB so far [16]. Here, the authors compared long BPL RYGB (150 cm BPL, AL 75 cm) with a short BPL RYGB (BPL 75 cm, AL 150 cm) in 128 patients and found a significant increase in %EWL for patients with long BPL RYGB 4 years after surgery. However, lengthening the BPL is not unlimited due to the increased risk of malnutrition [30]. Several studies suggest a minimal total alimentary limb length of 300 cm, which is defined as an added value of the AL and the CL, to reduce the risk of malnutrition with deficiencies of micro- and macronutrients [31–33].

Against this theoretical framework, it is difficult to draw conclusions on the optimal length of the AL and BPL, respectively, necessary to achieve the best possible outcome with a low risk of protein malnutrition. The most effective BPL length for the RYGB procedure has not been found yet since weight loss and improvement of comorbidities have only been evaluated in one non-randomized study [14] and one RCT [16].

As total bowel length varies by many meters between individuals, measuring the total length and therefore knowing the dimensions of all segments involved in RYGB will lead to a better understanding of the role of each segment in the beneficial effects after RYGB. This study investigates the effectiveness of long BPL RYGB compared to short BPL RYGB, analyzing defined clinical endpoints such as weight loss, morbidity and mortality, improvement in obesity-related comorbidities, and quality of life. Furthermore, the RCT by Homan et al. [16] failed to show any significant differences in T2DM remissions as it was only powered for weight loss. Our study has enough power to address the postoperative course in terms of resolution of comorbidities.

In conclusion, the “perfect” RYGB leads to adequate %TWL and improvement in obesity-related comorbidities without increasing the complication rate. The SLIM trial will answer questions about effectiveness and safety after long BPL RYGB and provide level I evidence for improvement of the standard RYGB. These findings might therefore potentially influence bariatric surgery guidelines on a global level.

## Trial status

Protocol version number 1.1, 12 February 2020. The trial has received ethics approval by January 2020. The first patient will be randomized in June 2020. We expect to enroll the calculated sample size in a 2- to 3-year time period. Estimated end of the study is December 2027.

## Supplementary Information

**Additional file 1.** SPIRIT 2013 Checklist: Recommended items to address in a clinical trial protocol and related documents.

## Data Availability

All data will be available for other research groups interested in conduction systematic reviews. Requests for data should be directed to the Sponsor Investigator, Ralph Peterli, ralph.peterli@clarunis.ch.

## Abbreviations

AL: Alimentary limb
BAROS: Bariatric analysis and reporting system
BMI: Body mass index
BPD: Biliopancreatic diversion
BPL: Biliopancreatic limb
CCI: Comprehensive Complication Index
CDC: Clavien-Dindo classification
CL: Common limb
EBMIL: Excess body mass index loss
eCRF: Electronic case report form
EWL: Excess weight loss
GIQLI: Gastrointestinal quality of life index
RYGB: Roux-en-Y gastric bypass
TALL: Total alimentary limb length
TWL: Total weight loss
